# 

**Published:** 1889-11-23

**Authors:** 


### NOTICE TO CORRESPONDENTS.

All MS., Letters, Books for Review, and other matters intended for the Editor, should be addressed The Editor, The Lodge, Porchester Square, London, W.

**Notice to Advertisers and Subscribers.**

All Advertisements, Orders for Copies of The Hospital, and Business Communications should be addressed to The Publisher, *not to the Editor*, The Hospital, 139-140, Salisbury-court, Fleet-street, E.C.

**Bound Volumes of "The Hospital."**

Vol. VI., containing full page portraits of T.R.H. the Prince and Princess of Wales, and Miss Florence Nightingale, is now ready, 4s., post free. Cases for binding, may be had of the Publisher, at 1s. 6d. each.

**To Residents, Secretaries, and Matrons**

The Editor will be glad to have the Regulations and each Report as issued by Asylums, Hospitals, Convalescent and Nursing Institutions, as well as those of other Charities, and an early copy in duplicate of all Appeals and Festival Notices, etc., addressed to *The Editor, The Lodge, Porchester-square, London, W.* Any superintendent, secretary, matron, or other chief official who regularly sends such information may be registered, on application to the Publisher, to receive free of cost a cover for binding The Hospital in half-yearly volumes.

### Scraps and Gleanings.

Typhus is prevalent at Horselydown.

Pneumonia is epidemic at Doncaster.

The body of the late Mr. Haynes Walton has been cremated.

Mr. Da Costa has been appointed surgeon-registrar to Guy's.

First aid to the injured is being taught in the Italian Military School.

Mr. Crerar, of Chicago, has left nearly a million of money to various charities.

Mr. W. Gibson has been appointed house surgeon at Burton-on-Trent Infirmary.

Messrs. Pears, of soap fame, have given £1,000 to the Newspaper Press Fund.

Dr. Oliver, of Preston Infirmary, lately submitted to transfusion of his blood to a patient.

Mr. T. A. Helme has been appointed resident medical officer at St. Mary's Hospital, Manchester.

Mr. E. E. Ware has been appointed house physician to the East London Hospital for Children.

The French Chamber of Deputies contains 47 M.D.'s, one officier de sante, one dentist, and one chemist.

Dr. Rubio, physician to the Queen of Spain, has been made a life Senator of the Spanish Government.

The Prince and Princess of Saxe-Weimar attended the bazaar at Kingstown in aid of the Monkstown Hospital.

Mr. C. E. H. Cotes, B.A., M.B., B.S., etc., has been elected surgeon to out-patients of Lock Hospital, Harrow-road.

On October 14th a committee met at the Carmarthenshire Infirmary, and passed the new rules after a few disputes.

Dr. Alice McLaughlin delivered the opening address at the winter session of the Women's Medical College, Toronto.

Dr. Panks, of Houghton, has been presented with £50 in recognition of his services during the late epidemic of typhoid.

Dr. Frankland says the waters of Loch Goil and Loch Long are not polluted by the Clyde Trust, but by local sewerage.

Great Britain contributed £1,334,491 to missionary work last year. This is an increase of £105,000 on the contributions of 1887.

Sir Watkin and Lady Williams-Wynn have been entertaining a large party at Wynnstay, for the Cottage Hospital Ball at Oswestry.

In the year ending October 31, 1,810 patients bitten by dogs were treated at the Pasteur Institute in Paris. There were thirteen deaths.

A learned Frenchman has published a treatise on "The Psychic Life of Micro-Organisms," which has just been translated into English.

Mr. John Henry Hunter, of Sheffield, has left £500 to the Sheffield General Infirmary, and £1,050 to other Sheffield charitable institutions.

The Charity Commissioners have postponed the consideration of the City of London charities till Jan. 15th, that they may hear from all the vestries.

Dr. Saymonne says he has discovered the cause of baldness to be a bacillus which invades the hair follicle and makes it brittle so that it breaks off.

Mr. John Harrold, assistant secretary to the London Fever Hospital, has been appointed secretary to the Royal Hospital for Diseases of the Chest, City-road.

The Lord Chief Justice is supporting the proposed Shaftesbury Hospital, which no physician or surgeon may attend who has ever held a license for vivisection.

There is considerable correspondence in the local papers about the proposed Blackpool Hospital. The inhabitants rather scoff at the notion of a cottage hospital.

Alderman J. G. Willis Willows, of Hull, has left £200 for the poor of Hessle, £100 to the Hull General Infirmary, £50 to the Hull Seaman's Orphan Asylum, and £50 to the Sculcoats Dispensary.

Miss E. M. Jowitt, late of Woodfield, near Leed, bequeathed legacies to religious and charitable societies to the amount of £5,650. Five distinctively women's societies received £1,500 between them.

A sleeping girl at Alaincourt, in France, is puzzling the physicians. Her slumber, though continuous, is not peaceful, as she appears to suffer from nightmare all the time. She is kept alive by Bouillon.

New Hospital for Women.—Three Cinderella dances will be given in aid of the maintenance fund. The first and second took place at the Westminster Town Hall on Thursday and Friday, the third will be given on February 6th.

Derbyshire Hospital for Sick Children treated during the year ending last June, 281 in-patients and 1,104 out-patients. Mr. Haslam has been elected president, and has given 100 guineas to the fund for purchasing more land.

At the French Cabinet Council it was decided to submit to the Council of State a decree authorising the Government to appropriate out of the legacies left to the State the sum of 400,000f. for the erection of a French hospital in Constantinople.

An extraordinary death has occurred at Chorley. A lodging-house keeper named James Meehan took a pinch of snuff, and began to sneeze immoderately. Blood issued from his mouth and nose, and he died before medical assistance could be obtained.

Bishop Barry preached last Monday at the anniversary festival of the Chapel of the Hospital for Sick Children, Great Ormond-street. He made a touching reference to the late Miss Ferguson, who organised the out-patient department of that institution.

The veterinary surgeons of the Madrid Municipality have detected a contagious disease which has been spreading rapidly among the milch cows used in Madrid and its suburbs. Strict instructions have been given to isolate and watch all cows so infected.

